# Managing possible serious bacterial infection of young infants where referral is not possible: Lessons from the early implementation experience in Kushtia District learning laboratory, Bangladesh

**DOI:** 10.1371/journal.pone.0232675

**Published:** 2020-05-11

**Authors:** Ahmed Ehsanur Rahman, Samantha Herrera, Sayed Rubayet, Goutom Banik, Rezaul Hasan, Ziaul Ahsan, Wahida Siraj, Anisuddin Ahmed, Abu Bakkar Siddique, Qazi Sadeq-ur Rahman, Lara M. E. Vaz, M. Jahurul Islam, M. Altaf Hossain, M. Shahidullah, M. Mohiuddin Osmani, Shams E. l. Arifeen, Stephen N. Wall

**Affiliations:** 1 International Centre for Diarrhoeal Disease Research Bangladesh (icddr,b), Dhaka, Bangladesh; 2 Save the Children, Saving Newborn Lives, Washington, DC, United States of America; 3 Ipas (formerly Save the Children International, Saving Newborn Lives), Dhaka, Bangladesh; 4 Save the Children International (formerly Saving Newborn Lives), Dhaka, Bangladesh; 5 Population Reference Bureau (formerly Save the Children, Saving Newborn Lives), Washington DC, United States of America; 6 National Newborn Health Program & Integrated Management of Childhood Illness (NNHP & IMCI), DGHS, MoHFW, Dhaka, Bangladesh; 7 Ministry of Health and Family Welfare (MoHFW), Dhaka, Bangladesh; 8 Bangabandhu Sheikh Mujib Medical University (BSMMU), Dhaka, Bangladesh; St George's University of London, UNITED KINGDOM

## Abstract

**Background:**

Serious infections account for 25% of global newborn deaths annually, most in low-resource settings where hospital-based treatment is not accessible or feasible. In Bangladesh, one-third of neonatal deaths are attributable to serious infection; in 2014, the government adopted new policy for outpatient management of danger signs indicating possible serious bacterial infections (PSBI) when referral was not possible. We conducted implementation research to understand what it takes for a district health team to implement quality outpatient PSBI management per national guidelines.

**Methods:**

PSBI management was introduced as part of the Comprehensive Newborn Care Package in 2015. The study piloted this package through government health systems with limited partner support to inform scale-up efforts. Data collection included facility register reviews for cases seen at primary level facilities; facility readiness and provider knowledge and skills assessments; household surveys capturing caregiver knowledge of newborn danger signs and care-seeking for newborn illness; and follow-up case tracking, capturing treatment adherence and outcomes. Analysis consisted of descriptive statistics.

**Results:**

Over the 15-month implementation period, 1432 young infants received care, of which 649 (45%) were classified as PSBI. Estimated coverage of care-seeking increased from 22% to 42% during the implementation period. Although facility readiness and providers’ skills increased, providers’ adherence to guidelines was not optimal. Among locally managed PSBI cases, 75% completed the oral antibiotic course and 15% received the fourth day follow-up. Care-seeking remained high among private providers (95%), predominantly village health doctors (over 80%).

**Conclusions:**

Facility readiness, including health care provider knowledge and skills were strengthened; future efforts should focus on improving provider adherence to guidelines. Social and behavior change strategies targeting families and communities should explore shifting care-seeking from private, possibly less-qualified providers. Strategies to improve private sector management of PSBI cases and improved linkages between private and public sector providers could be explored.

## Introduction

Possible serious bacterial Infections (PSBI) or syndromic sepsis is a global public health concern for newborns and young infants (0–59 days), with an estimated 30 million cases per year [1, 2]. Severe infections including sepsis, pneumonia, meningitis, and tetanus, account for around a quarter of all neonatal deaths globally [3–5]. PSBI in newborns and young infants is of significant concern, particularly for Africa and South Asia, with inadequate service readiness and compromised quality of care [6–12].

Up until 2015, the World Health Organization (WHO) recommended inpatient hospital care for all young infants with PSBI and treatment with multi-drug multi-dose injectable antibiotics (penicillin or ampicillin and gentamicin) for 7–10 days [13]. Every child with PSBI, according to the guideline, should have been referred from a first-level facility to a referral level, in-patient facility for management. In most low-resource settings, however, this guideline of referral was not feasible for families or the health system, with studies finding that referrals were not possible for approximately 80% of such infants [14, 15]. The multi-country Simplified Antibiotic Therapy Trials (SATT) in South Asia (Bangladesh and Pakistan) and the African Neonatal Sepsis Trial (AFRINEST) in Democratic Republic of Congo, Kenya, and Nigeria, tested and demonstrated the safety and efficacy of simplified antibiotic regimens for treating PSBI in young infants [16–18] in outpatient settings. Based on the evidence from the SATT and AFRINEST studies, WHO issued a new guideline in 2015 for management of PSBI cases when referral was not possible [19]. The revised guideline still recommends admitting young infants with PSBI for inpatient care. However, in cases where referral is not feasible or accepted, the revised guideline recommends treating PSBI cases—without sign(s) of critical illness—through outpatient care with simplified antibiotic regimens consisting of fewer doses of injectable antibiotics, along with oral antibiotics that can be administered at home [19].

Bangladesh is one of 25 Countdown countries that achieved the Millennium Development Goal 4 target [20, 21]. Despite this progress, declines in neonatal mortality have lagged and the country’s neonatal mortality rate remains high [22]. Reducing mortality due to PSBI is critical for Bangladesh, as it accounts for more than one-third of all newborn deaths [23]. Given the high PSBI burden and the country’s commitment to reach the 2030 Sustainable Development Goal neonatal mortality rate target of 12 or below per 1,000 live births, in 2014, the Bangladesh National Technical Working Committee for Newborn Health (NTWC-NH) formed a special working group to review the SATT and AFRINEST trial findings in order to develop a strategy for Bangladesh. The committee recommended that sick young infants with signs of PSBI be managed with a simplified antibiotic regimen at first-level primary care facilities in Bangladesh, where referral is not possible [16–18, 24–26]. In response, the Government of Bangladesh (GoB)—with technical support from Save the Children’s Saving Newborn Lives (SNL) Program and evaluation support from icddr,b—conducted implementation research in Kushtia District to assess the feasibility of implementing the recommended simplified antibiotic regimen in a real-life setting, explore operational challenges, and identify potential solutions and lessons to inform national scale-up.

### Adoption of PSBI guidelines within Bangladesh’s comprehensive newborn care package

In 2013, the GoB, through its declaration of “A Promise Renewed: Child Survival Call to Action,” committed to the adoption and national scale-up of five new evidence-based newborn health interventions: 1) management of sick young infants with PSBI in primary level care facilities; 2) chlorhexidine application for prevention of cord infection (as part of essential newborn care); 3) antenatal corticosteroids for prevention of complications from preterm labor; 4) kangaroo mother care for small babies; and 5) specialized newborn care units at the upazila (sub-district) and district level [27, 28]. In 2014, a Comprehensive Newborn Care Package (CNCP) was developed incorporating these five new interventions as part of the National Newborn Health Strategy; the treatment regimens adopted in 2014 by the NTWC-NH for PSBI where referral is not accepted or feasible were incorporated within the CNCP. With technical support from SNL, the GoB developed national guidelines for outpatient management of young infants with PSBI.

**Fig 1:**
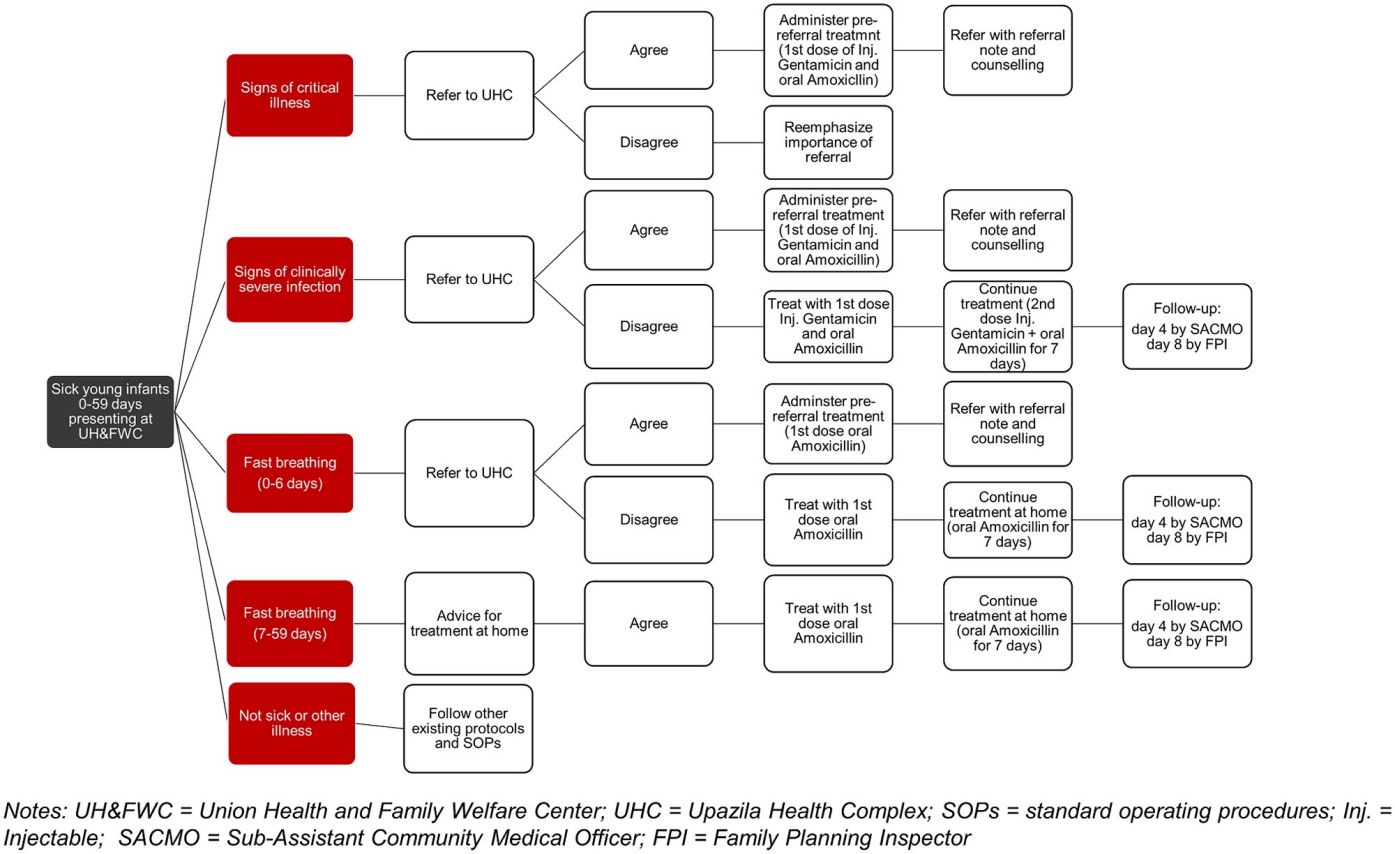
Algorithm of the Bangladesh national PSBI case management guidelines where referral is not accepted.

PSBI in young infants has three sub-classifications: 1) critical illness (CI), 2) clinical severe infection (CSI), and 3) isolated fast breathing (IFB) (Refer to text box for clinical definitions). National treatment algorithms, referral, and follow-up of cases are detailed in Fig 1. CI cases identified at primary care level facilities are to be administered pre-referral treatment and then referred to a higher-level facility for inpatient care. Similarly, CSI cases are to be referred and, if referral is accepted, provided pre-referral treatment. If referral is not accepted or feasible, the cases are to be treated at the primary care facility with two doses (once daily for first 2 days) of injectable gentamicin and oral amoxicillin twice daily for 7 days. IFB cases among young infants aged 0–6 days are to be referred and, if referral is not feasible, the cases are to be treated with oral amoxicillin twice daily for 7 days. IFB cases among young infants aged 7–59 days do not need to be referred and can be treated with oral amoxicillin twice daily for 7 days. Follow-up of cases managed at the primary care facility level is expected on day 4 through a telephone call and on day 8 through a home visit.

Union Health and Family Welfare Centers (UH&FWCs), primary care facilities at the union level, were selected to serve as the delivery point for outpatient PSBI case management and Upazila Health Complexes (UHCs) as the referral facility. UH&FWCs serve populations of 25,000–35,000 and have a Sub-Assistant Community Medical Officer (SACMO), who has 3 years of clinical training and provides general curative care; most SACMOs have received training in Integrated Management of Childhood Illness.

Management of young infants with PSBI at UH&FWCs is carried out by SACMOs, which includes conducting a telephone follow-up of the young infant on day four. Lay field supervisors, called Family Planning Inspectors (FPI), are tasked with conducting follow-up home visits on day eight for PSBI cases managed at the UH&FWC.

### Implementation of the CNCP district learning lab in Kushtia

The Ministry of Health and Family Welfare’s (MOHFW) Directorate General of Health Services (DGHS) and Directorate General of Family Planning (DGFP) selected Kushtia District as a demonstration and learning site to implement the CNCP (hereinafter referred to as a district learning lab), since the district was not receiving any external donor support for maternal and newborn health at the time and the need for newborn care services was high. The purpose of the district learning lab was to demonstrate, using limited external resources and technical support, how the CNCP, and specifically the new interventions, could be effectively and sustainably scaled-up at district level within the existing public health structure using local resources and budgets. While the aim of this work was to document and share learning to inform the national scale-up of the full CNCP, the focus of this paper is on the learning from the implementation research of management of young infants with PSBI.

The CNCP was formally launched in Kushtia District in early 2015, covering the district’s six upazilas (Bheramara, Daulatpur, Kumarkhali, Kushtia Sadar, Khoksa, and Mirpur). SNL provided technical support to DGHS and DGFP for implementation start-up, with activities focused on: advocacy and policy support for improving national and district-level readiness for PSBI case management; building the capacity of the health care providers in PSBI case management; improving health facility readiness; monitoring and supervision of implementation to inform program adaptation; and promoting the use of newborn care services at UH&FWCs through community awareness and engagement. Implementation of PSBI case management in UH&FWCs began in October 2015.

#### Advocacy and policy support for improving national and district-level readiness for PSBI case management

SNL provided advocacy and technical support to the MOHFW to facilitate necessary policy changes for implementation of the new guidelines for PSBI case management at UH&FWCs. This support included technical assistance in the development and dissemination of the national guideline on PSBI case management. SNL helped advocate for a policy change to allow SACMOs working under DGFP to administer injectable gentamicin. In addition, the DGFP drug list was updated to include injectable gentamicin, which previously was only available through the DGHS. PSBI commodity distribution guidelines were also developed for health care managers and providers.

#### Capacity building of health care providers in PSBI case management

Capacity building focused on knowledge and skills development and retention. Formal training on the national guidelines for PSBI case management was provided as part of the CNCP training for different health care provider cadres. The SACMOs received a five-day CNCP training that included a half-day on identification and management of PSBI. In addition, SACMOs also received a one-day orientation on the sick newborn and young infant service register, reporting forms, and two jobs aids (PSBI management algorithm and dosage chart, and the injection administration and sepsis case follow-up chart). Mini skills labs were set up with SNL support at UHCs to facilitate on-site practice of provider skills in PSBI recognition and management, with mock practice sessions conducted during monitoring visits and at monthly SACMO meetings. On-the-job mentoring was provided to SACMOs by upazila level managers, who served as mentors and provided advice and feedback during routine visits to facilities. FPIs received a four-day training covering counseling and awareness on newborn danger signs, community awareness and engagement responsibilities, and provision of follow-up care for young infants with PSBI who receive outpatient treatment.

#### Health facility readiness for PSBI management

SNL provided support to the DGHS and DGFP to strengthen the readiness of the UH&FWCs, specifically to ensure availability of newborn medicines, equipment, supplies, and logistics. Given CNCP was introducing new interventions, drugs and commodities required for management of young infants with PSBI were not initially on the MOHFW procurement list. As an interim measure, SNL procured and distributed commodities (injectable gentamicin, oral amoxicillin, and insulin syringes) through existing government distribution structures to all the UH&FWCs in Kushtia. SNL also provided the district hospital, UHCs, and UH&FWCs with the necessary initial equipment (baby weighing machines, thermometers, and acute respiratory infection (ARI) timers) for management of young infants with PSBI. An orientation and follow-up technical support was provided to district and upazila level storekeepers and pharmacists on newborn-related logistics management, including support to strengthen the reporting system on monthly consumption. The MOHFW, through the DGHS and DGFP, began procuring and distributing commodities for PSBI in June 2017.

#### Monitoring and supervision of implementation

Several monitoring and supervision tools were developed with technical support from SNL. A sick newborn and young infant service register and reporting forms were developed to record and monitor PSBI case management and commodity stocks at the facility level. A PSBI surveillance reporting form was developed for the FPIs to use to track follow-up home visits on day eight. Additionally, a referral form was introduced to strengthen the linkage between the UH&FWCs and referral facilities (UHC) for PSBI case management, and a UH&FWC monitoring checklist was developed to guide supervisory visits (see S1 Table for details on the register and reporting forms developed).

Monthly data on PSBI cases managed and stock availability at the UH&FWCs were submitted to and aggregated at the upazila level. Regular supervision and monitoring were conducted primarily using existing mechanisms. A medical officer from the civil surgeon office at the district level and medical officers at the upazila level were appointed to monitor the CNCP implementation (inclusive of PSBI case management). District and upazila level managers carried out routine facility visits to UH&FWCs, where they monitored PSBI case management activity, checked stock and equipment status, reviewed registers and reporting forms, and assessed provider knowledge retention. SNL staff accompanied upazila managers on supervisory visits to build capacity; and, after approximately a year of implementation, gradually shifted away from providing this support. These supervisory visits were guided by the UH&FWC monitoring checklist to ensure all components were addressed during visits. Observations from these supervisory visits were discussed in routine monthly upazila level staff meetings. Additionally, quarterly one-day review meetings were organized for SACMOs to review registers, analyze data on PSBI cases, and assess and address gaps in provider adherence to the national guidelines. Quarterly meetings also served as an opportunity to identify and address data reporting issues and errors.

#### Community awareness of newborn health care services

As part of CNCP, a social and behavior change (SBC) campaign was developed and implemented in Kushtia. For PSBI, the campaign focused on increasing awareness of newborn danger signs and promotion of early care-seeking at UH&FWCs. SBC approaches included:

- Education and awareness raising through routine contacts with government community health workers (e.g. home visits, Expanded Program on Immunization sessions) and during visits to health facilities. As part of the CNCP training, health care providers at community, union, and upazila levels, were oriented on and provided with different SBC materials for counseling clients during antenatal, delivery, and postnatal care contacts (e.g. flip sheets, flip charts, and posters).
- Community education to improve knowledge of newborn danger signs and early care-seeking at UH&FWCs. Community groups and community support groups received an orientation on the CNCP and were provided with educational materials (a flip sheet and a booklet) to use during group sessions.
- TV spots aired through local cable networks on essential newborn care and newborn danger signs, and posters on newborn danger signs were placed in the waiting areas of health facilities (district hospital, UHCs, and UH&FWCs).

A summary of the different implementation activities, inputs, and the timeline for the district learning lab in Kushtia can be found in the S1 Table.

## Materials and methods

### Study context and settings

This study was implemented in Kushtia District, approximately 200 kilometers west of Dhaka, Bangladesh’s capital. At the time of the study, the neonatal mortality rate in Kushtia was 36 deaths per 1,000 live births, slightly higher than the national average of 32 deaths per 1,000 live births [29] (unpublished data). In consultation with the MOHFW, three of the six CNCP learning lab upazilas of Kushtia—Daulatpur, Mirpur, and Kumarkhali—were selected for the study. These were three of the largest upazilas in Kushtia, with the exception of Kushtia Sadar upazila, which was not selected because it did not have a UHC but rather was served directly by the Kushtia District Hospital (see S2 Table for more details on the selected upazilas).

Ten unions were selected from each upazila, in consultation with local health managers and SNL. Seven unions in the three upazilas were excluded because they were difficult to access or for other logistical reasons. The study was conducted in all available 36 UH&FWCs in the 30 selected unions.

### Study objectives

The main objective of this study was to assess changes in the utilization and quality of case management of young infants with signs of PSBI, per the National Sepsis Management Guidelines, in the selected UH&FWCs in Kushtia district. Quality of care for PSBI case management was assessed in terms of health facility readiness, adherence to guidelines by health care providers and caregivers, and immediate outcomes (treatment failure). A secondary objective of the study was to assess changes in caregiver knowledge of newborn danger signs and in care-seeking for newborn illness.

### Study design

We employed the following quantitative methods of data collection: repeated rounds of health facility readiness assessment surveys; repeated rounds of health care provider knowledge and skill assessment surveys; continuous data extraction from the UH&FWC sick newborn and young infant service registers; follow-up interviews with caregivers of sick young infants who received care at UH&FWCs; and repeated rounds of household surveys in the selected unions. The study was implemented over a 15-month period (divided into 5 quarters), between December 2015 and February 2017; in addition, select pre and post-implementation assessments were conducted. Table 1 summarizes the data collection methods and their respective timelines for this study.

**Table 1:** Data collection timeline by data collection method.

Data Collection Method		Study implementation period (December 2015–February 2017)					
	Pre	Q1	Q2	Q3	Q4	Q5	Post
Health facility assessment surveys	R1	R2		R3	R4		R5
Knowledge and skill assessment surveys		R1		R2			R3
Data extraction from registers*		X	X	X	X	X	
Follow-up household interviews		X	X	X	X	X	
Household surveys		X	X	X	X	X	

Q = Quarter, R = round.

*Data extraction from registers was conducted weekly throughout the study period.

### Study population

The study population included young infants with signs of PSBI receiving care from the selected UH&FWCs and their primary caregivers, health care providers in the selected UH&FWCs responsible for identifying and treating PSBI, and women with a recent history of childbirth in the selected unions.

### Sampling strategy and sample size

For the health facility assessment surveys, all UH&FWCs in the selected unions were approached for assessment. Of the 36 UH&FWCs in the 30 unions, 29, 30, 34, 34, and 34 were assessed across the five rounds of surveys, respectively; not all 36 UH&FWCs could be assessed in every round due to non-availability of SACMOs.

For the knowledge and skill assessments, three rounds of surveys were conducted of 30, 31, and 34 SACMOs, respectively. The rounds of knowledge and skill assessments were independent of the rounds of health facility assessments.

Data extraction was conducted weekly from the newborn and young infant service registers from the selected UH&FWCs throughout the study implementation period (December 2015–February 2017). A total of 1440 records for young infants receiving care with signs of PSBI and other illness were extracted over the 15-month period, of which 1432 were less than 60 days of age on the day of their health facility visit.

Follow-up visits and household interviews with primary caregivers were attempted between 7–14 days after the initial UH&FWC visit, for all sick young infants identified from the UH&FWC registers. A total of 1243 follow-up visits were completed, 1087 within the target 7–14 day window.

For the household survey, a stratified sampling technique was used to ensure equal representation of all selected unions from the three upazilas. Villages were considered as the primary sampling unit. Probability proportional to size sampling was used to select 17 villages from each union. From the selected villages, all women with a recent history of childbirth (in the three months preceding the survey) were identified. A total of 4436 women were interviewed through four rounds of surveys during the study period.

### Data collection instruments and procedures

The health facility assessments were conducted by trained study physicians using a structured assessment tool that had specific sections on service availability and readiness for PSBI case management. The knowledge and skill assessment surveys were conducted on SACMOs by trained study physicians with a structured interview questionnaire and checklist. The tool had specific questions on knowledge regarding the PSBI classification and management guidelines, and a skills assessment section that presented different hypothetical case scenarios.

The data extraction was conducted by trained data collectors using a structured extraction tool. All variables available in the sick newborn and young infant service registers (presenting signs, classifications, treatment received from UH&FWCs, and status of follow up), were extracted. The follow-up visits were conducted in the households of sick young infants by trained study nurses using a structured questionnaire. The study nurses conducted a clinical assessment to assess the condition of the young infants on the day of the visit and interviewed primary caregivers regarding care-seeking practices and adherence to treatment advice.

The household survey was conducted by trained data collectors with an interviewer-administered structured questionnaire. The questionnaire included questions on background characteristics and sociodemographic information, caregiver knowledge of newborn danger signs, and care-seeking practices.

### Data analysis methods

Descriptive statistics were used to report readiness of UH&FWCs for PSBI case management, knowledge and skills of SACMOs, utilization of UH&FWCs by young infants, adherence to treatment protocol by SACMOs and treatment advice by caregivers, treatment failure rates, knowledge of caregivers regarding newborn danger signs, and care-seeking by caregivers for sick young infants. Changes in these indicators are presented by quarter.

Health facility readiness was scored based on the availability of 10 essential items for managing PSBI according to the national guidelines. These items included oral amoxicillin, injectable gentamicin, insulin syringes, baby weighing machine, thermometer, ARI timer, sick newborn and young infant service registers, prescription with referral slip, and two job aids (a dose calculation table for antibiotics for PSBI management and a visible algorithm for PSBI management). The items were selected based on consultation with clinical experts and national health managers. Facility readiness was categorized as poor (0–4 items), moderate (5–7 items), and good (8–10 items) based on their availability. Information on functionality was collected for baby weighing machines, thermometers, and ARI timers in three of the five assessment rounds.

Coverage at UH&FWCs of young infants with PSBI presenting for care was estimated, assuming a population incidence of 9.5 PSBI cases per 100 young infants [30], then dividing the number of cases seen in the facility each quarter with the estimated number of PSBI cases in the catchment population for the UH&FWCs. Catchment population in 2016 (mid-point between December 2015 and February 2017) was estimated from the 2010 census report, after adjusting for annual growth rate. The annual birth cohort of a union was estimated assuming 19 births per 1,000 population [31].

Adherence to treatment was calculated based on caregiver recall of receiving injectable and oral drugs at UH&FWCs and giving oral antibiotics at home, collected through the follow-up interviews with caregivers. Treatment failure was defined as the presence of any danger sign, including signs of CI, CSI, and IFB, as assessed by study nurses during the follow-up household visits conducted on days 7–14.

Knowledge of caregivers regarding newborn danger signs was calculated based on 14 items (Annex 1). Any woman who could mention (unprompted) four or more danger signs was considered to have good knowledge.

Data were analyzed using Stata 14.0 (StataCorp. 2015. Stata Statistical Software: Release 14. College Station, TX: Stata Corp LP).

### Quality control and assurance

The data collectors received extensive training by the study investigators before starting data collection. Biweekly refresher trainings were held to discuss data quality issues. Field supervisors conducted regular spot checks to assess the quality of data collection. Additionally, monthly supervisory visits and random spot checks were conducted by study investigators. Finally, all filled-in questionnaires were reviewed by field supervisors and field managers before data entry.

### Ethical approval and consent to participate

Ethical approval for the study was obtained from the Institutional Review Board of icddr,b (Protocol Number: PR 15074) and the Save the Children Ethics Research Committee in the United States. Written informed consent was obtained from all participants before beginning interviews. Privacy, anonymity, and confidentiality of the participants were strictly maintained during data collection and analysis.

## Results

### Health facility readiness for PSBI case management

**Fig 2:**
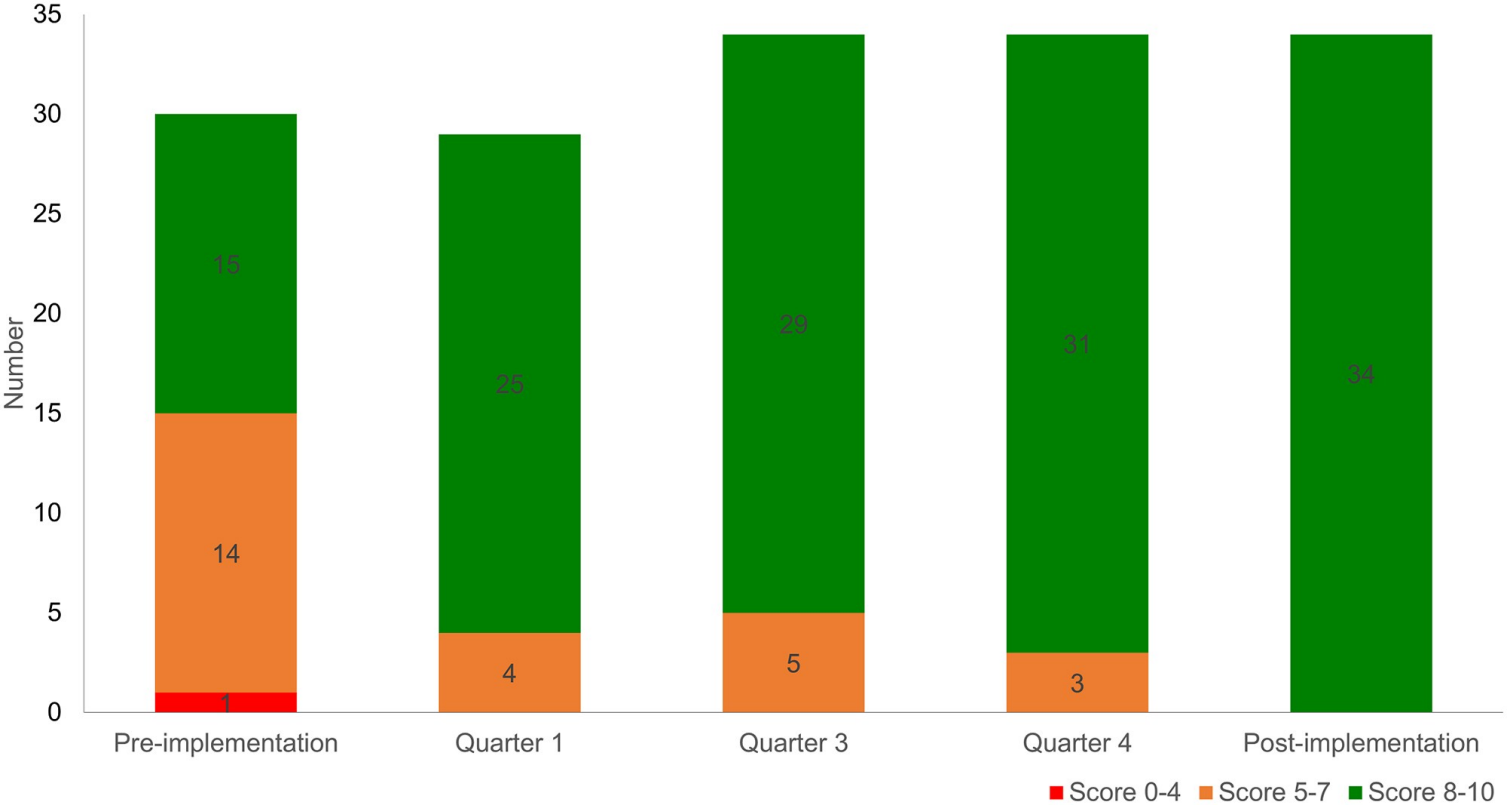
Number of UH&FWCs by readiness score for management of sick young infants with PSBI, by quarter, 2015–2017 (health facility assessment surveys, N: Pre-implementation = 30, quarter 1 = 29, quarters 3, 4 and post-implementation = 34).

UH&FWCs readiness for management of young infants with PSBI is presented in Fig 2. In the pre-implementation phase, only half of the facilities had at least eight of the 10 essential items available. Readiness improved by quarter 1 and remained stable: around 80% of assessed facilities had at least eight items available in quarters 1, 3 and 4. In the post-implementation phase, all facilities had at least eight items available. Of the 10 items, oral amoxicillin and ARI timers were the most common items unavailable during the different rounds of assessments (see S3 Table). The availability of oral amoxicillin was the most variable during the 15-month study period: 79% in quarter 1, 73% in quarter 3, and 53% in quarter 4; at post-implementation it was assessed at 97%. Availability of ARI timers was less than 20% through quarter 3, then improved substantially in quarter 4 (94%) and the post-implementation period (100%). Functionality of ARI timers, thermometers, and baby weighing machines was assessed in three of the assessment rounds (pre-implementation, quarter 2 and 3) (S1 Fig); overall, there were no major issues during the two assessments in the study implementation period, although there were some facilities that reported functionality issues with the weighing machines in quarter 2 (5 of 29 facilities).

### Health care provider skills in PSBI assessment

Overall, health care providers’ skills in PSBI assessment improved from the beginning of implementation (quarter 1) through the post implementation phase (S2 Fig). The largest improvements were observed in assessment for central cyanosis (from 23% to 71%), skin pustules (from 50% to 91%), bulging fontanelle (from 33% to 71%), and weighing and recording the infant’s weight (from 37% to 77%). There was an improvement in the assessment of respiratory rate between quarter 1 and 3 (from 43% to 97%), but then a decline observed in the post implementation assessment (50%).

### Utilization of UH&FWCs for sick young infants

**Fig 3:**
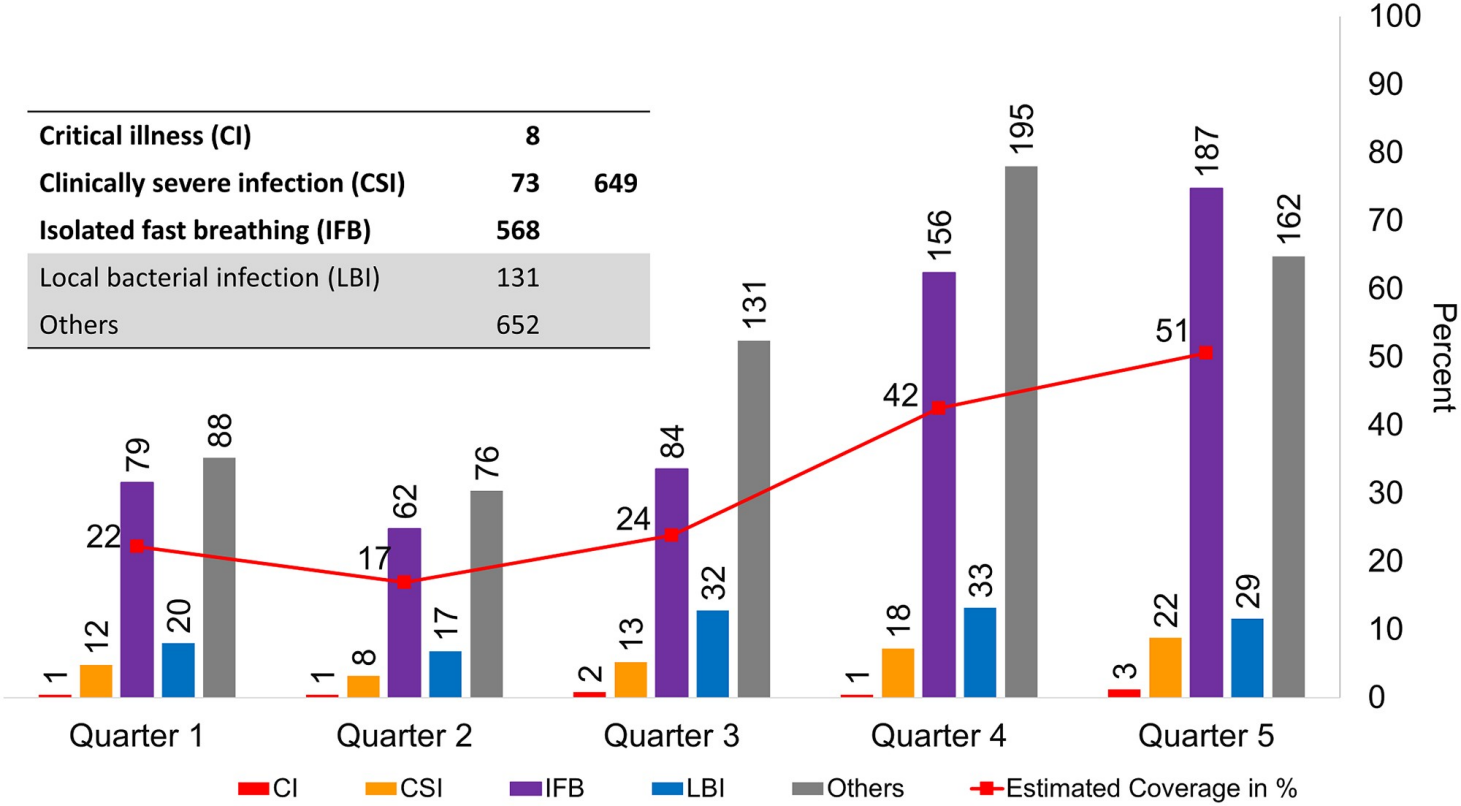
Number of sick young infants, including PSBI cases, receiving care from UH&FWC facilities and estimated coverage of expected PSBI cases (percent), from health facility registers, December 2015-February 2017 (N = 1432).

Fig 3 summarizes the utilization of UH&FWCs by sick young infants. A total of 1432 sick young infants of age less than 60 days received care from 36 first level facilities between December 2015 and February 2017. Of them, 649 were classified as PSBI—eight as critical illness (CI), 73 as clinically severe infection (CSI), and 568 as isolated fast breathing (IFB); 131 cases had local bacterial infection (LBI) and 652 cases were classified as other. Utilization of UH&FWCs for CI remained low and unchanged throughout the assessment period. The number of CSI cases at the UH&FWCs increased slightly across the five quarters, but likewise remained relatively low. The number of IFB cases showed a gradual increase from 79 in quarter 1 to 187 in quarter 5. Fig 3 also presents estimated coverage of PSBI cases managed. The estimated case management coverage of PSBI cases increased gradually from 22% in quarter 1 to 51% in quarter 5, with an estimated coverage of 31% over the 15-month period; the largest increase was observed between quarter 3 and quarter 4 (from 24% to 42%). Half of the health facilities (18 of 36 UH&FWCs) treated 10 or fewer sick young infants with PSBI, 25% treated 11–30 cases, 17% treated 31–50 cases, and 8% treated 51 or more PSBI cases between December 2015 and February 2107 (S3 Fig).

### Health care provider and caregiver adherence to PBSI guideline and treatment outcomes

**Fig 4:**
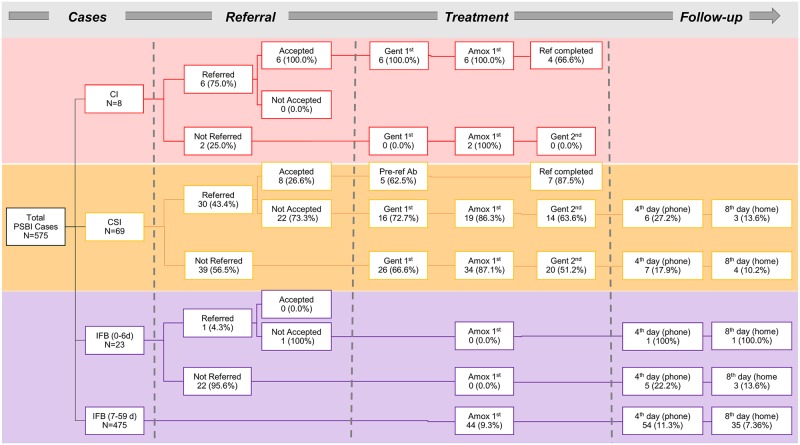
Providers’ adherence to guidelines for management of sick young infants with PSBI in UH&FWC facilities, (data source: Caregiver report from community case tracking, N = 575).

Fig 4 presents the health service providers’ adherence to guidelines, based on primary caregiver reports during case tracking. Of the 649 PSBI cases, 575 (88%) were successfully tracked through the community follow-up visits. These included eight classified as CI, 69 as CSI, 23 as IFB 0–6 days, and 475 as IFB 7–59 days. Of the eight CI cases, six caregivers reported being referred to higher-level facilities for inpatient care. All six reported accepting referral and receiving appropriate pre-referral treatment (first dose of injectable gentamicin and oral amoxicillin). The two other CI cases reported that they were not referred and received the first dose of oral amoxicillin only; no doses of injectable gentamicin were reported to have been given. Among the 69 CSI cases, caregivers of only 30 reported being referred and eight accepted the referral. Out of these eight cases, only five received appropriate pre-referral treatment (first dose of injectable gentamicin and oral amoxicillin). Out of those who did not accept the referral (n = 22), 16 reported receiving the first dose of injectable gentamicin, 19 reported receiving the first dose of amoxicillin at the health facility, and 14 reported receiving the second dose of injectable gentamicin. Only six reported receiving the fourth day follow-up phone call from the provider, and three reported receiving the eighth day follow-up home visit. Similarly, among the CSI cases who were not offered to be referred (n = 39), caregivers of 26 reported that the young infant received the first dose of injectable gentamicin, 34 reported receiving the first dose of amoxicillin at the health facility, and 20 reported receiving the second dose of injectable gentamicin. Only seven reported receiving the fourth day follow-up phone call from the provider, and four reported receiving the eighth day follow-up home visit. Of the 23 IFB 0–6 day cases identified in the facilities, none received the first dose of oral amoxicillin before leaving the facility and only six and four received the fourth and eighth day follow-ups, respectively. Of the 475 cases classified as IFB 7–59 days, less than 10% received the first dose of oral amoxicillin before leaving the facility, only 11% and 7% received the fourth and eighth day follow-ups, respectively.

**Fig 5:**
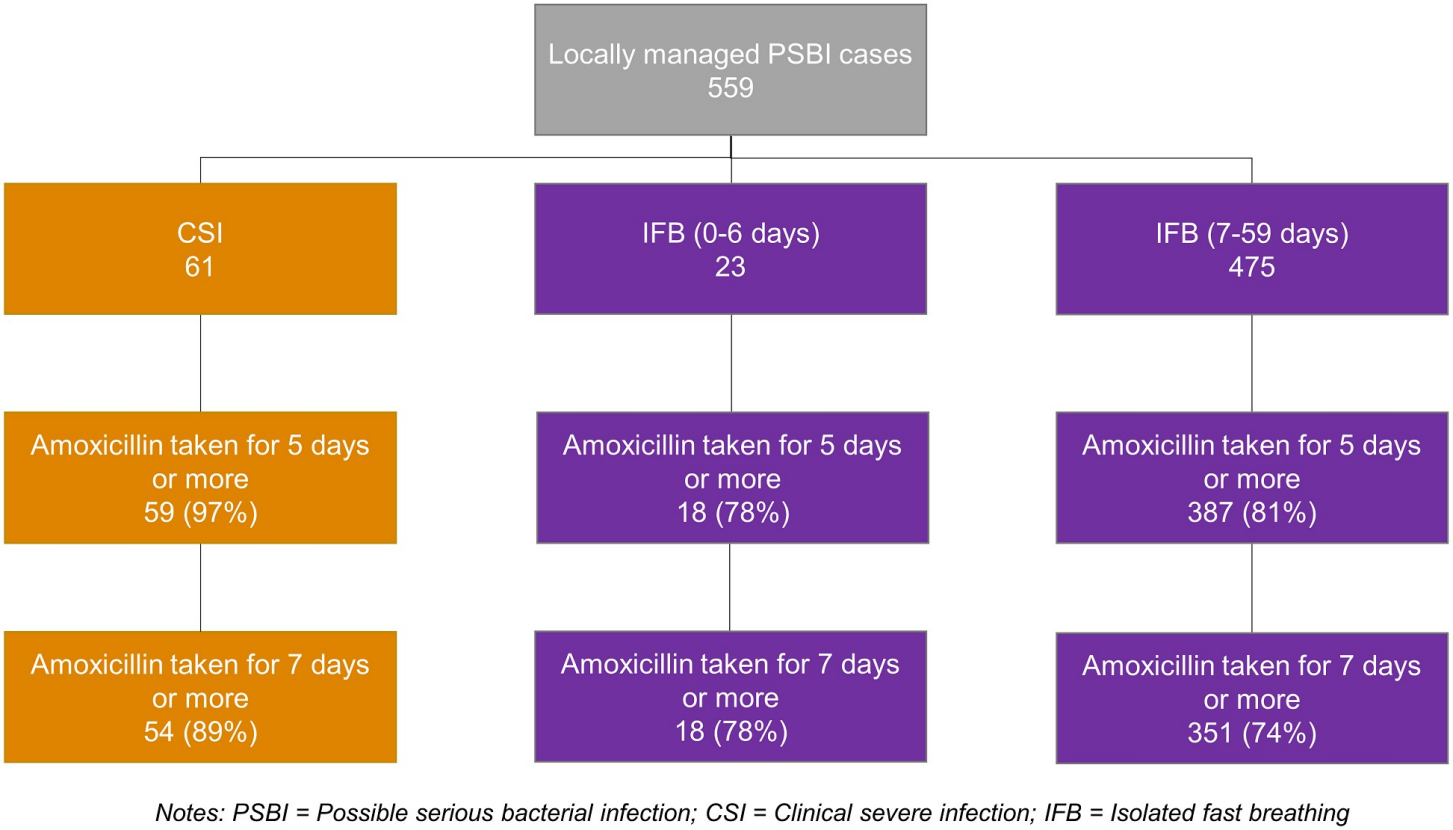
Caregiver report of adherence to treatment of sick young infants with PSBI (source: Community case tracking, N = 559).

Fig 5 presents the caregivers’ adherence to the treatment provided for locally managed PSBI cases. Of the 61 CSI cases, 97% of caregivers reported that their infant had received oral amoxicillin for five days and 89% for seven days. Of the IFB cases (498), approximately 80% of caregivers reported that their infant had received oral amoxicillin for five days and 74% for seven days. The treatment failure rate was 0% for both locally managed CSI and IFB 0–6 days cases, and approximately 5% among IFB 7–59 days cases. For the 2 CI cases who were not referred, the caregivers reported that their infant did not receive any gentamicin but received oral amoxicillin for at least five days (not presented in Fig 5). No deaths were reported among the CI, CSI, and IFB cases managed at UH&FWCs where community case tracking occurred (S4 Fig).

### Women’s knowledge of newborn danger signs and care-seeking for newborn illness

Background characteristics of women with a recent history of birth in the three months preceding the survey are presented in the S4 Table. No notable difference was observed in any of the background characteristics across the five quarters.

Women’s knowledge of newborn danger signs improved gradually over the 15-month implementation period, from 28% in quarter 1 to 42% in quarter 5 (S5 Fig). Across all five quarters, the majority (over 90%) of the women reported seeking care for their sick young infant (see S5 Table). Most care was sought in the private sector (overall 85%), ranging from 81% to 88% across the 15-month study period; only 10% of caregivers across the surveys sought care at a public sector facility. The most common source of care sought was village doctors (overall, 56%), followed by private hospitals or clinics (overall, 14%). Overall, only 3% of caregivers reported seeking care at a UH&FWC; a small increase was observed from 1.5% in quarter 1 to 5% in quarter 5.

## Discussion

This study examined the feasibility of implementing the national guidelines for PSBI case management as part of the CNCP in Kushtia, with a focus on assessing the utilization and quality of PSBI case management in primary level UH&FWCs in the district. The study showed that UH&FWC readiness for PSBI case management gradually improved over the implementation period, with a substantial improvement observed between pre-implementation and the first quarter of implementation. Health system inputs provided to the UH&FWCs were targeted to ensure the essential elements required for PSBI case management were available. Initial equipment and commodity inputs were provided by the program at the beginning of implementation to help ensure health facility readiness and then gradually this role was transitioned to the GoB. The post-implementation results show all facilities with a higher score on the readiness index, indicating that health facility readiness for PSBI case management was sustained through the local health system structures. Similar improvements in the knowledge and skills of SACMOs in PSBI case management were observed across the three rounds of health care provider assessments, despite the low caseload managed by providers (half of the UH&FWCs managed fewer than 10 cases in the 15-month implementation period). These results suggest that the capacity building and skill retention strategies implemented in Kushtia helped to sustain provider theoretical knowledge and skills around assessment and treatment for PSBI.

Quarterly data on sick young infant cases managed at UH&FWCs (from the data extraction records) showed a gradual increase in cases seen over the 15-month implementation period; however, most of the increase was for less severe infections (IFB and LBI) and for cases classified as other. The number of CI cases seen at UH&FWCs remained very low over the implementation period, and CSI cases only showed a small increase in the last two quarters. The household survey data similarly showed a small increase in care-seeking for sick young infants at UH&FWCs (from 1.5% in quarter 1 to 5% in quarter 5). Estimates of PSBI case incidence in young infants 0–59 days from the recent Aetiology of Neonatal Infection in South Asia (ANISA) study show incidence at 9.5% [30]; using these estimates, we calculated that approximately 31% of the expected number of PSBI cases were managed at UH&FWCs in Kushtia over the 15-month period. Low utilization was also shown in other districts in Bangladesh (11.2%) [32]. Overall, coverage in Kushtia was generally lower than other implementation research sites in sub-Saharan Africa and South Asia which ranged from 58%-97% [33]; however, coverage in these other sites included cases presenting in hospitals, whereas in our study we report only those that reported to first-level public facilities.

Multiple factors likely contributed to low utilization of UH&FWCs for care of sick young infants. The CNCP introduced in Kushtia included new interventions not historically offered in the UH&FWCs. While community awareness and demand generation strategies were included in the overall CNCP strategy as a means to improve awareness of newborn danger signs and care-seeking for newborn health services at UH&FWCs, they may have not been sufficient to build awareness and observe substantive changes in care-seeking. Data on the reach and quality of implementation of these strategies were not systematically collected in the study. However, programmatic documentation indicates that implementation of the community awareness activities may have lagged and not been implemented consistently in all sites, suggesting there likely was insufficient time and activities for greater changes in care-seeking to be observed. Data from the household surveys did show a modest increase in caregiver knowledge of newborn danger signs (from 28% in quarter 1 to 42% in quarter 5) and a small increase in care-seeking from UH&FWCs, indicating some improvement over the implementation period (S5 Table). While the UH&FWCs were selected as the point of service delivery due in part to their widespread availability, there is also a high availability of informal and private sector providers in the area [34]. For many families, private sector and informal providers, particularly local village doctors, are widely available and likely to be closer to home in terms of proximity [35, 36] and many studies have shown caregiver preference for these providers [37–40]. Data from the household surveys showed that care-seeking from the private and informal providers, particularly local village doctors, remained high (above 80% across all survey rounds) throughout the implementation period (Annex 1). Altogether, the findings indicate modest changes in care-seeking for sick young infants at UH&FWCs toward the latter part of implementation, but that preference for private and informal sector providers remained high. Given these findings, effective strategies for shifting care-seeking to qualified providers may warrant further exploration.

Health care provider adherence to the guidelines for managing PSBI cases, assessed through caregiver report, was not optimal. While the number of CI cases managed at the UH&FWCs was small, only six of the eight were referred and provided the proper pre-referral treatment. Among CSI cases managed by SACMOs at the UH&FWCs, only 69% of caregivers reported having received the first dose of injectable gentamicin and just 56%, the two required doses. For IFB cases, only 64% of caregivers reported receiving amoxicillin from the health care provider for treatment at home. Overall, health care provider adherence to the treatment protocol in Kushtia appears to have been lower than other study sites in sub-Saharan Africa (all above 90%) and South Asia (Pakistan, 81%) [33]. Project documentation highlighted challenges with caregiver acceptance of referral; noting issues such as distance to the referral facility, lack of money, the need for a guardian to accompany the caregiver and child to the referral facility, among others. Providers’ understanding of these challenges faced by families may have influenced their adherence to the protocol on referral of cases. A few logistical challenges to the administration of the second dose of injectable gentamicin by providers were also documented. UH&FWCs are closed on the weekends, which can disrupt the course of treatment on the second day, and competing responsibilities of the SACMOs can take them away from the UH&FWCs (e.g. school health education sessions), also affecting treatment provision. Health facility readiness data also indicated there were some stockouts of injectable gentamicin and amoxicillin (S3 Table); it is possible other disruptions in supply occurred during the implementation period that affected provider adherence to the treatment protocol. Among the caregivers that reported receiving amoxicillin, adherence to provision of amoxicillin for the full 7 days was generally high, at 89% and 74%, for CSI and IFB cases, respectively. These results suggest that the majority of caregivers are being provided with proper counseling on treatment administration at home and are adhering to the advice.

The caregiver interviews indicate that the majority of PSBI cases that were supposed to be referred per the national guideline were not (57% of CSI cases; and 96% of IFB cases in infants 0–6 days). The majority of cases that reported having received a referral also reported not accepting the referral, instead choosing to have the infant treated at the UH&FWC. Reasons for the low adherence to the protocol by providers are not clear; given the results are based on the caregiver report, there is the possibility of recall or social desirability bias. The exception was the CI cases, where the majority (six of eight) reporting being referred and accepting referral.

Provider adherence to the follow-up protocol in Kushtia was substantially lower than in other study sites, which reported follow-up adherence of 90% or higher [33]; it is possible that the other study sites implemented study-specific follow-up protocols, whereas in Kushtia the follow-up was per national guidelines and with existing public sector staff only. Among CSI and IFB cases managed locally, only 13% (out of 559 cases) of caregivers reported receiving a follow-up call on the fourth day and 8% reported receiving a home visit by the FPI on the eighth day after the initial visit to the UH&FWC. Several challenges in adhering to the follow-up protocol were voiced by health care providers during implementation. The fourth day follow-up via phone with caregivers was perceived to be challenging. In some cases, caregivers were unaware of their phone numbers and thus unable to provide a number for the provider to call; some caregivers gave the number of their husband or neighbor, resulting in the provider not always being able to reach the primary caregiver of the newborn; in other cases, the provided phone number would be turned off. Due to these challenges, the protocol shifted during implementation to encourage caregivers to call or visit the UH&FWC on the fourth day for follow-up, instead of having the follow-up be initiated only by the provider. Program documentation also highlighted challenges in completing the eighth day follow-up, including issues in the coordination of the follow-up visit between the SACMO and the FPI, and FPIs incorporating the follow-up visits into their existing workloads. In some of the sites, FPIs were responsible for covering more than one union due to worker vacancies/shortages, which resulted in greater distances and workloads to cover. At present, only the fourth day follow-up visit is recorded in the UH&FWC register, a gap that will need to be addressed in order to adequately monitor adherence to the full follow-up protocol in the future.

Treatment failure, measured as the presence of any PSBI signs or symptoms between 7 and 14 days, was minimal in the study. Among CI, CSI, and IFB cases managed locally, treatment failure was observed in 3.9% of cases, all among IFB cases. No deaths were reported in the 15-month implementation period among cases seen in UH&FWCs. Treatment failure in other study sites have ranged from less than 1% to 12% [33, 41, 42]; thus, treatment failure was relatively lower in Kushtia. The lower rate could be because the majority of cases treated were less severe (mainly IFB) or that families sought care for more severe cases at other facilities.

Overall, the results suggest that while improvements and retention of SACMOs knowledge and skills in PSBI case management were measured, these are not necessarily translating into actual practice and adherence to the national guidelines by providers. This could be in part due to the logistical challenges noted, including caregiver inability to adhere to referral, drug stockouts, issues with functionality of equipment, and challenges in conducting follow-up visits. However, given the overall poor adherence observed, this should be further explored to better characterize the key barriers to improve provider adherence to PSBI guidelines and identify potential solutions. The study findings also showed care-seeking among private and informal providers, particularly from local village doctors, remained high throughout the implementation period. While modest changes were observed in care-seeking at public facilities, the findings suggest that the CNCP SBC strategy and its implementation should be revisited to influence care-seeking behavior. Moreover, future efforts should also explore possible engagement of the private sector to improve sick young infant treatment for PSBI.

## Limitations

Completion of the community follow-up interviews with caregivers of sick young infant cases was challenging; only 87% (1243 of 1432 cases) of cases identified at the UH&FWCs were successfully followed-up after the initial visit at the UH&FWC, and only 76% (1087 of 1432 cases) of the cases received a follow-up visit within the desired 7–14 day timeframe. Given provider and caregiver adherence to the national guidelines, and treatment failure were assessed during the follow-up interviews with caregivers, it is possible that cases not followed-up were different from those that were followed-up, potentially biasing the results. Furthermore, provider adherence to PSBI guidelines was determined based on caregiver recall, and was therefore subject to recall error. While data extracted from facility registers could have provided additional insight on protocol adherence and treatment follow-up, problems with completeness and proper completion limited their usability. Given the low caseload of CI and CSI cases, results on health provider and caregiver adherence to the treatment protocol and treatment failure were presented only for the full 15-month implementation period rather than by quarter, limiting the study’s ability to assess possible changes in treatment adherence and treatment failure over time.

## Conclusions

This paper presents the key findings and lessons from the early implementation experience of managing young infants with PSBI at primary level health facilities in Kushtia District. Findings show some improvements over the implementation period in health facility readiness for PSBI case management and in provider skills to provide PSBI case management, as well as caregiver knowledge of newborn danger signs and relatively good adherence to treatment advice. However, despite the various inputs provided and approaches used to improve and sustain health care provider knowledge and skills for PSBI case management, provider adherence to the national guideline was below optimal. Future efforts to implement the guidelines need to explore factors influencing provider behaviors. Finally, for higher effective coverage of outpatient management of PSBI when referral is not feasible, SBC strategies targeting families and communities need to be fine-tuned to shift care-seeking from private, possibly less-qualified providers. Alternately, strategies to improve private sector management of PSBI cases and improved linkages between private and public sector providers may need to be explored.

## Supporting information

S1 Appendix Definitions. (DOCX)

S1 Table Summary of Kushtia district learning lab intervention activities, inputs, details, and timeframe. (DOCX)

S2 Table Characteristics of selected upazilas and available health facilities, 2014. (DOCX)

S3 Table Percentage of UH&FWCs with availability of the 10 essential items for PSBI case management, by quarter. (DOCX)

S4 Table Background characteristics of women with a history of recent birth, by quarter. (DOCX)

S5 Table Caregiver report of care-seeking for young infants in the two weeks preceding the survey, by quarter and overall. (DOCX)

S1 Fig Number of UH&FWCs with essential equipment available and functional over time for management of infants with PSBI, by quarter, 2015–2017. (TIF)

S2 Fig Percent of UH&FWC providers (SACMOs) demonstrating skills to manage PSBI cases, by quarter. (TIF)

S3 Fig Distribution of first level facilities by number of sick young infants with PSBI managed between Dec 2015 –Feb 2017. (TIF)

S4 Fig Treatment failure among locally managed PSBI cases. (TIF)

S5 Fig Percent of women with a recent history of birth with knowledge (unprompted) of 4 or more newborn danger signs, by quarter. (TIF)

S1 Dataset Community case tracking dataset. (DTA)

S2 Dataset Data extraction from registers dataset. (DTA)

S3 Dataset Health facility assessment survey dataset. (DTA)

S4 Dataset Household survey dataset. (DTA)

S5 Dataset Knowledge and skill assessment of SACMOs dataset. (DTA)
