# Supplementary material for: Managing possible serious bacterial infection of young infants where referral is not possible: Lessons from the early implementation experience in Kushtia District learning laboratory, Bangladesh
Source: PLoS One. 2020 May 11;15(5):e0232675. doi: 10.1371/journal.pone.0232675 (PMC7213695; doi:10.1371/journal.pone.0232675)
Supplement: S1 Appendix — (DOCX) [file pone.0232675.s001.docx]

# **S1 Appendix**

**Definitions**

**Caregiver’s knowledge assessment regarding newborn danger signs**

The following 14 newborn danger signs were used to assess the knowledge level of the caregivers in the household surveys:

1. Not feeding well
2. Persistent vomiting
3. Vomits everything out or projectile vomiting
4. Poor or no sucking reflex
5. Low temperature (hypothermia)
6. Central cyanosis
7. Fever or raised temperature
8. Respiratory rate greater than 60 breaths per minute
9. Severe chest in-drawing
10. Very difficult breathing
11. Less movement than normal or movement only when stimulated
12. Unconscious or drowsy
13. Convulsions or history of convulsions
14. Pus or foul smelling discharge from umbilicus; or red and swollen umbilicus

**Health facility readiness assessment**

The health facility readiness were assessed based on the availability of the following 10 items:

1. Oral amoxicillin (pediatric amoxicillin drop)
2. Injectable gentamycin
3. Thermometers
4. Acute respiratory infection (ARI) timers
5. Insulin syringe
6. Baby weighing machine
7. Sick Newborn and Young Infant Service Register
8. Prescription with referral slip
9. Dose Calculation Table-Antibiotics for Sepsis Management
10. Visible Algorithm for Sepsis Management

Categorization of health facility readiness:

*Poor= Availability of 0-4 items*

*Moderate = Availability of 5-7 items*

*Good = Availability of 8-10 items*

**Definition of Possible Severe Bacterial Infection (PSBI)**

Presence of any of the following signs in young infants:

**Critical Illness (CI)**

- Unconscious or drowsy
- Convulsions or history of convulsions
- Unable to feed
- Persistent vomiting
- Bulging fontanelle
- Central cyanosis
- Weight less than 1500 gm or very low birth weight

**Clinical Severe Infection (CSI)**

- Severe chest in-drawing
- Temperature less than 96°F (hypothermia)
- Fever or temperature more than 99.5°F
- Not feeding well
- Less movement than normal or movement only when stimulated

**Isolated Fast Breathing (IFB)**

- Respiratory rate greater than 60 breaths per minute

**Definition of treatment failure**

Presence of any of the following sings in young infants during the date of follow up visit (within 7-14 days of initial visit to facility):

**Critical Illness (CI)**

- Unconscious or drowsy
- Convulsions or history of convulsions
- Unable to feed
- Persistent vomiting
- Bulging fontanelle
- Central cyanosis
- Weight less than 1500 gm or very low birth weight

**Clinical Severe Infection (CSI)**

- Severe chest in-drawing
- Temperature less than 96°F (hypothermia)
- Fever or temperature more than 99.5°F
- Not feeding well
- Less movement than normal or movement only when stimulated

**Isolated Fast Breathing (IFB)**

- Respiratory rate greater than 60 breaths per minute
