# Supplementary material for: Managing possible serious bacterial infection of young infants where referral is not possible: Lessons from the early implementation experience in Kushtia District learning laboratory, Bangladesh
Source: PLoS One. 2020 May 11;15(5):e0232675. doi: 10.1371/journal.pone.0232675 (PMC7213695; doi:10.1371/journal.pone.0232675)
Supplement: S1 Table — (DOCX) [file pone.0232675.s002.docx]

**S1 Table.** Summary of Kushtia District Learning Lab intervention activities, inputs, details, and timeframe

| **Intervention Area** | **Activities** | **Specific Inputs/Details** | **Timeframe** |
| --- | --- | --- | --- |
| National readiness for implementation of outpatient management of possible severe bacterial infection (PSBI) | Development of the national guidelines for PSBI case management | Technical insight and assistance provided by the Saving Newborn Lives Program (SNL) | 2014 – 2015 |
|  | Update the Ministry of Health and Family Welfare’s Directorate General of Family Planning (DGFP) policy to allow Sub-Assistant Community Medical Officers (SACMOs) to administer injectable gentamicin | - Technical assistance provided by SNL to review and update SACMO roles and responsibilities in job description and role in integrated management of childhood illness program - SNL advocacy with professionals, program managers and NTWC-NBH members to agree on PSBI management by SACMO | February 2016 |
|  | Advocacy and technical support for development of PSBI commodity distribution guidelines | Technical assistance provided by SNL | April 2014 |
|  | Advocacy to update the DGFP drug list to include injectable gentamicin | - Technical advocacy provided by SNL for inclusion of PSBI commodities on agenda of the Newborn Technical Working Committee (NTWC) meeting - Technical assistance provided by SNL to include injectable gentamicin in DGFP drug list | February 2016 |
| Capacity building at district level of health care providers* in PSBI case management | Comprehensive Newborn Care Package (CNCP) training for:   - Doctors and senior staff nurses (SSN) working at five Upazila Health Complexes (UHCs) and Kushtia District Hospital - SACMOs and Family Welfare Visitors (FWVs) working at 64 Union Health & Family Welfare Centers (UH&FWCs) and 5 UHCs | Five-day CNCP health provider training provided to:   - 81 Doctors - 188 SSN - 129 SACMOs (64 of which work at the UH&FWCs) - 64 FWVs | April – May 2015 |
|  | Training on CNCP for community health workers and supervisors | Four-day CNCP community health worker training provided to 859 community health workers across the six cadres of providers and supervisors | June – July 2015 |
|  | Two-day CNCP refresher training for select health care providers (doctors, SSN, FWVs) and community health workers to ensure all health care providers received training on CNCP and to support skill retention | Two-day CNCP refresher training provided to:   - 105 doctors - 184 SSN - 65 FWVs - 634 CHWs | October – December 2016 |
|  | Mini skills lab sessions and mock practice with SACMOs to support skill retention conducted during:   - Monthly SACMO meetings at UHCs - Monitoring visits conducted at UH&FWCs | SNL provided technical support, materials and equipment to set up mini skills labs at UHCs | Early 2016 – onwards |
| Improvement of health facility readiness | Procurement and provision of equipment for PSBI case management (baby weighing machines, thermometers, and acute respiratory infection [ARI] timers) | With support from SNL, procured and supplied 64 UH&FWCs, 5 UHCs and the district hospital with:   - Baby weighing machines - Thermometers - ARI timers | December 2014 |
|  | Procurement of drugs and commodities for PSBI case management | Procurement by SNL of oral amoxicillin, injectable gentamicin, and insulin syringes to:   - 64 UH&FWCs - 5 UHCs (to ensure sufficient stores of stock) | April 2015 – June 2017 |
|  | Orientation on newborn logistics management provided for district and upazila level storekeepers and pharmacists | Two orientation sessions held with storekeepers and pharmacists | June 2015, April 2017 |
|  | Technical support for logistics reporting form for newborn commodities | Logistics reporting form developed and provided to storekeepers at district and upazila levels | September 2015 |
|  | Capacity development around newborn logistics | - Orientation meetings with storekeeper staff at district and upazila levels - Technical assistance provided by SNL to include logistics issues discussion in monthly meeting agenda - Storekeeper participation in monthly meetings to address supply chain issues | February 2016, April 2017 |
| Monitoring and supervision of implementation | Development and distribution (through the GoB supply distribution system) of registers and reporting forms for facility- and community-level follow-up:   - Sick newborn and young infant service register for case management by SACMOs - PSBI case surveillance form for community follow-up visits by FPIs - Union level sick newborn and young infant management monthly reporting form, to compile monthly PSBI case data - Upazila level monthly compilation report form on sick newborn and young infant management - Union and upazila stock registers | - For health care providers at UH&FWCs (SACMOs): Sick newborn and young infant services registers and the monthly reporting forms that include PSBI commodities stock data - For community supervisors (FPIs): Registers for PSBI case surveillance and community follow-up of PSBI cases managed at the UH&FWCs - For upazila level (Upazila Family Planning Assistants): Monthly sick newborn and young infant management compilation forms, rolling up information from union level | Registers and reporting forms for health care providers at UH&FWCs and UHCs: September 2015  Register for FPIs: November 2015 |
|  | Training on registers and reporting forms | - 64 SACMOs trained on sick newborn register and monthly reporting form - 55 FPIs trained on surveillance form | September 2015 (SACMOs)  November 2015 (FPIs) |
|  | Supervisory visits by national, district, and upazila level managers to UH&FWCs | - Technical inputs by SNL to develop a UH&FWC monitoring checklist for supervisor visits - Upazila and district health managers and SNL conducted joint supervisory and monitoring visits using monitoring check list - Supervisory visits conducted at least once per quarter, with poor performing facilities receiving additional supervisory visits. | Ongoing from November 2015  *November 2015 - November 2016: Joint supervisory visits by SNL and local health managers, afterwards conducted by local health managers only* |
|  | Quarterly review meetings with health managers from DGHS and DGPF for SACMOs | 8 quarterly review meetings held; initially at district level and then later organized for each upazila (sub-district) | June 2015 – July 2017 |
| Community awareness of sick newborn services** | Orientation and refresher for community groups (CGs) and community support groups (CSGs) | Initial orientation and refresher provided to:   - 198 CG members - 594 CSG members | 1^st^ Orientation:  September – October 2015  Refresher:  July – September 2016 |
|  | Development and provision of job aids and health education materials for community health workers on newborn danger signs (flip-sheet) | - Technical inputs by SNL on jobs aids and health education materials - Job aids and education materials printed and distributed through the Government of Bangladesh (GoB) supply system to all community health care providers and their respective supervisors (FWAs, HAs, FPIs, AHIs, HIs and CHCPs) | April 2015 |
|  | Provision of job aids and health education materials for health care providers at the facility level on newborn danger signs and newborn health services (flip charts, posters) | Supplied job aids and health education materials for facility level health care providers who are responsible for provision of antenatal, delivery, and/or postnatal care services (doctors, SSN, SACMOs, midwives, and FWVs) at:   - 64 UH&FWCs - 5 UHCs - 1 district hospital | April 2015 |
|  | Provision of health education materials on newborn danger signs and newborn health services for community group members (flip-sheet and booklet) | - 198 CGs - 594 CSGs | April 2015 |
|  | Mass media and small media on newborn health services (television commercials, posters) | - Television commercials on essential newborn care and newborn danger signs aired on local cable channels - Three posters developed on birth preparedness, essential newborn care, and newborn danger signs; placed in the waiting areas of public sector facilities (district hospital, 5 UHCs, 64 UH&FWCs, and 198 community clinics) | TV commercials: September 2015 – June 2017 (aired daily)  Posters: September 2015 |

Notes: * Includes community health workers (FWVs and FPIs) and facility-based health care providers (doctors, nurses, SACMOs). **Orientation and training of health care providers at the UHCs and UH&FWCs on social and behavior change materials was included in the CNCP training, captured under the “capacity building of health care providers in PSBI case management”.
