# Supplementary material for: Managing possible serious bacterial infection of young infants where referral is not possible: Lessons from the early implementation experience in Kushtia District learning laboratory, Bangladesh
Source: PLoS One. 2020 May 11;15(5):e0232675. doi: 10.1371/journal.pone.0232675 (PMC7213695; doi:10.1371/journal.pone.0232675)
Supplement: S2 Table — (DOCX) [file pone.0232675.s003.docx]

**S2 Table.** Characteristics of selected upazilas and available health facilities, 2014

| **Upazila** | **Area**  **km^2^** | **Population (2011)** | **Number of** | | | **Number of OPD patients (2014)** | | | |
| --- | --- | --- | --- | --- | --- | --- | --- | --- | --- |
|  |  |  | **Unions** | **USC** | **UH&FWC** | **UHC** | | **USC** | |
|  |  |  |  |  |  | **all ages** | **U5** | **all ages** | **U5** |
| Daulatpur | 472.5 | 522,027 | 14 | 14 | 10 | 46,557 | 5,330 | 58,188 | 10,330 |
| Kumarkhali | 258.18 | 340,215 | 11 | 7 | 6 | 40,277 | 12,010 | 80,609 | 12,399 |
| Mirpur | 317.35 | 346,807 | 12 | 12 | 11 | 27,230 | 9,967 | not available | not available |

Data Source: Local Health Bulletin, DGHS, Bangladesh. Notes: OPD = outpatient department; USC = union sub center; UH&FW = union health and family welfare centers; UHC = upazila health centers; U5 = children under age five.
