# Supplementary material for: Managing possible serious bacterial infection of young infants where referral is not possible: Lessons from the early implementation experience in Kushtia District learning laboratory, Bangladesh
Source: PLoS One. 2020 May 11;15(5):e0232675. doi: 10.1371/journal.pone.0232675 (PMC7213695; doi:10.1371/journal.pone.0232675)
Supplement: S3 Table — (DOCX) [file pone.0232675.s004.docx]

**S3 Table.** Percentage of UH&FWCs with availability of the 10 essential items for PSBI case management, by quarter

| **Essential Item** | | **Pre** | **Quarter 1** | **Quarter 3** | **Quarter 4** | **Post** |
| --- | --- | --- | --- | --- | --- | --- |
|  |  | **%** | **%** | **%** | **%** | **%** |
| 1 | Oral amoxicillin | 70.0 | 79.3 | 73.5 | 52.9 | 97.1 |
| 2 | Injectable gentamicin | 86.7 | 100.0 | 100.0 | 91.2 | 97.1 |
| 3 | Insulin syringe | 100.0 | 100.0 | 97.1 | 94.1 | 100.0 |
| 4 | Baby weighing machine | 100.0 | 96.6 | 100.0 | 91.2 | 100.0 |
| 5 | Thermometer | 66.7 | 65.5 | 79.4 | 91.2 | 100.0 |
| 6 | ARI timer | 13.3 | 17.2 | 14.7 | 94.1 | 100.0 |
| 7 | Sick newborn and young infant service registers | 80.0 | 100.0 | 100.0 | 100.0 | 100.0 |
| 8 | Prescription with referral slip | 86.7 | 89.7 | 97.1 | 100.0 | 100.0 |
| 9 | Job aid 1 (antibiotics dose calculation table) | 86.7 | 100.0 | 100.0 | 100.0 | 100.0 |
| 10 | Job aid 2 (visible algorithm for PSBI management) | 93.3 | 100.0 | 100.0 | 100.0 | 100.0 |
| **Total UH&FWCs** | | **30** | **29** | **34** | **34** | **34** |

Data source: Health facility assessment surveys. Notes: Pre = pre-implementation phase; Post = post-implementation phase.
