# Supplementary material for: Managing possible serious bacterial infection of young infants where referral is not possible: Lessons from the early implementation experience in Kushtia District learning laboratory, Bangladesh
Source: PLoS One. 2020 May 11;15(5):e0232675. doi: 10.1371/journal.pone.0232675 (PMC7213695; doi:10.1371/journal.pone.0232675)
Supplement: S4 Table — (DOCX) [file pone.0232675.s005.docx]

**S4 Table.** Background characteristics of women with a history of recent birth, by quarter

| Background Characteristic | | Quarter 1  % | Quarter 2 % | Quarter 3 % | Quarter 4 % | Quarter 5 % |
| --- | --- | --- | --- | --- | --- | --- |
| Sex of the baby | Male | 49.4 | 51.7 | 49.7 | 50.9 | 50.2 |
|  | Female | 50.6 | 48.3 | 50.3 | 49.1 | 49.8 |
| Mother’s age | 15-19 | 21.8 | 17.8 | 19.5 | 21.8 | 20.6 |
|  | 20-24 | 33.0 | 33.3 | 34.9 | 31.3 | 34.3 |
|  | 25-29 | 28.2 | 29.2 | 29.5 | 28.7 | 26.0 |
|  | 30+ | 16.9 | 19.7 | 16.1 | 18.2 | 19.1 |
| Mother’s education level | No education | 8.5 | 8.6 | 6.1 | 7.4 | 6.2 |
|  | Primary incomplete | 7.9 | 7.9 | 5.3 | 6.8 | 6.2 |
|  | Primary complete | 13.0 | 13.3 | 15.3 | 13.4 | 15.3 |
|  | Secondary incomplete | 47.2 | 49.6 | 46.7 | 51.3 | 49.4 |
|  | Secondary complete or higher | 23.4 | 20.5 | 26.5 | 21.0 | 22.9 |
| Mother's profession | No work | 90.4 | 94.2 | 95.3 | 93.0 | 94.8 |
|  | Physical work | 8.2 | 4.2 | 3.7 | 6.6 | 4.1 |
|  | Nonphysical/professional | 1.4 | 1.5 | 1.0 | 0.4 | 1.1 |
| Father’s age | 15-19 | 0.5 | 0.7 | 0.3 | 0.2 | 0.1 |
|  | 20-24 | 10.6 | 11.6 | 8.3 | 12.2 | 10.6 |
|  | 25-29 | 32.9 | 28.1 | 32.5 | 31.3 | 32.4 |
|  | 30-34 | 26.9 | 28.4 | 31.9 | 25.1 | 28.6 |
|  | 35-39 | 17.5 | 19.1 | 17.2 | 20.8 | 17.8 |
|  | 40+ | 11.0 | 12.1 | 9.8 | 10.4 | 10.4 |
|  | Don’t know | 0.5 | 0.7 | 0.3 | 0.2 | 0.1 |
| Father’s education level | No education | 26.9 | 29.1 | 24.5 | 25.9 | 23.3 |
|  | Primary incomplete | 8.7 | 8.5 | 6.9 | 7.2 | 6.1 |
|  | Primary complete | 13.0 | 15.3 | 16.0 | 18.0 | 18.4 |
|  | Secondary incomplete | 24.8 | 23.2 | 22.7 | 23.6 | 23.1 |
|  | Secondary complete or higher | 25.8 | 23.6 | 29.6 | 24.8 | 28.9 |
|  | Don’t know | 0.7 | 0.2 | 0.3 | 0.4 | 0.2 |
| Upazila | Daulatpur | 39.6 | 44.2 | 50.0 | 42.7 | 40.9 |
|  | Kumarkhali | 36.0 | 22.9 | 20.3 | 24.0 | 30.5 |
|  | Mirpur | 24.4 | 32.9 | 29.7 | 33.3 | 28.6 |
| Religion | Muslim | 98.8 | 98.6 | 99.1 | 98.2 | 98.8 |
|  | Non-Muslim | 1.2 | 1.4 | 0.9 | 1.8 | 1.2 |
| Wealth Quintile | Lowest | 21.1 | 22.8 | 18.2 | 17.4 | 19.2 |
|  | Second | 19.3 | 21.9 | 17.4 | 23.2 | 20.1 |
|  | Middle | 19.6 | 18.4 | 22.2 | 19.8 | 19.8 |
|  | Fourth | 20.1 | 19.2 | 20.1 | 20.8 | 20.0 |
|  | Highest | 19.9 | 17.7 | 22.0 | 18.6 | 20.9 |
| Total N |  | **1289** | **848** | **976** | **499** | **824** |

Data source: Repeated household surveys
