# Supplementary material for: Managing possible serious bacterial infection of young infants where referral is not possible: Lessons from the early implementation experience in Kushtia District learning laboratory, Bangladesh
Source: PLoS One. 2020 May 11;15(5):e0232675. doi: 10.1371/journal.pone.0232675 (PMC7213695; doi:10.1371/journal.pone.0232675)
Supplement: S5 Table — (DOCX) [file pone.0232675.s006.docx]

**S5 Table.** Caregiver report of care-seeking for young infants in the two weeks preceding the survey, by quarter and overall

|  | **Quarter 1** | **Quarter 2** | **Quarter 3** | **Quarter 4** | **Quarter 5** | **Overall** |
| --- | --- | --- | --- | --- | --- | --- |
|  | **%** | **%** | **%** | **%** | **%** | **%** |
| **Caregiver sought care** |  |  |  |  |  |  |
| Yes | 94.8 | 96.7 | 94.6 | 93.6 | 94.9 | 95.0 |
| No | 5.2 | 3.3 | 5.4 | 6.3 | 5.1 | 5.0 |
| *Total N* | *423* | *335* | *390* | *234* | *353* | *1,735* |
| **Source of care by sector *** |  |  |  |  |  |  |
| Private sector* | 80.8 | 87.0 | 87.5 | 84.9 | 83.9 | 84.7 |
| Public sector | 10.7 | 9.0 | 8.9 | 10.5 | 9.9 | 9.8 |
| NGO sector | 0.5 | 0.0 | 0.5 | 0.5 | 0.9 | 0.5 |
| Other | 8.0 | 4.0 | 3.0 | 4.1 | 5.4 | 5.0 |
| *Total N* | *401* | *324* | *369* | *219* | *335* | *1,648* |
| **Source of care by type** |  |  |  |  |  |  |
| Village doctor | 48.4 | 59.9 | 60.4 | 57.1 | 57.9 | 56.4 |
| Private hospital or clinic | 17.2 | 13.9 | 14.1 | 13.2 | 10.5 | 14.0 |
| MBBS doctor chamber | 6.2 | 7.1 | 6.0 | 5.5 | 6.3 | 6.3 |
| Paramedic/MA/SACMO | 4.2 | 4.6 | 4.6 | 6.9 | 8.4 | 5.6 |
| Other facility | 7.7 | 4.0 | 3.0 | 4.1 | 5.4 | 5.0 |
| UH&FWC | 1.5 | 2.2 | 1.9 | 4.1 | 5.1 | 2.8 |
| UHC | 4.0 | 1.9 | 2.7 | 1.4 | 1.5 | 2.4 |
| Allopathic drug store | 4.5 | 1.5 | 1.9 | 2.3 | 0.9 | 2.3 |
| Community clinic | 0.8 | 2.8 | 1.1 | 3.2 | 1.2 | 1.6 |
| District hospital | 1.5 | 1.2 | 1.6 | 0.9 | 1.2 | 1.3 |
| MCWC | 2.5 | 0.6 | 1.1 | 0.5 | 0.6 | 1.2 |
| Other sources** | 1.5 | 0.3 | 1.6 | 0.8 | 1.0 | 1.1 |
| *Total N* | *401* | *324* | *369* | *219* | *335* | *1,648* |

Data source: Repeated household surveys. Notes: *Private sector providers include private hospital and clinics, private health center, MBBS doctor chamber, village doctor, private sector paramedic/medical assistant/sub-assistant community medical officer, allopathic drug store, other private provider. **Includes other sources that individually were all less than 1%: medical college hospitals, other public sector facilities, NGO hospital or satellite clinic, and private health centers. MBBS = Bachelor of Medicine, Bachelor of Surgery; MA = medical assistant; SACMO = sub-assistant community medical officer; UH&FWC = Union Health and Family Welfare Center; UHC = Upazila Health Center; MCWC = Maternal and Child Welfare Center.
