# Supplementary figures and images for: Managing possible serious bacterial infection of young infants where referral is not possible: Lessons from the early implementation experience in Kushtia District learning laboratory, Bangladesh
Source: PLoS One. 2020 May 11;15(5):e0232675. doi: 10.1371/journal.pone.0232675 (PMC7213695; doi:10.1371/journal.pone.0232675)

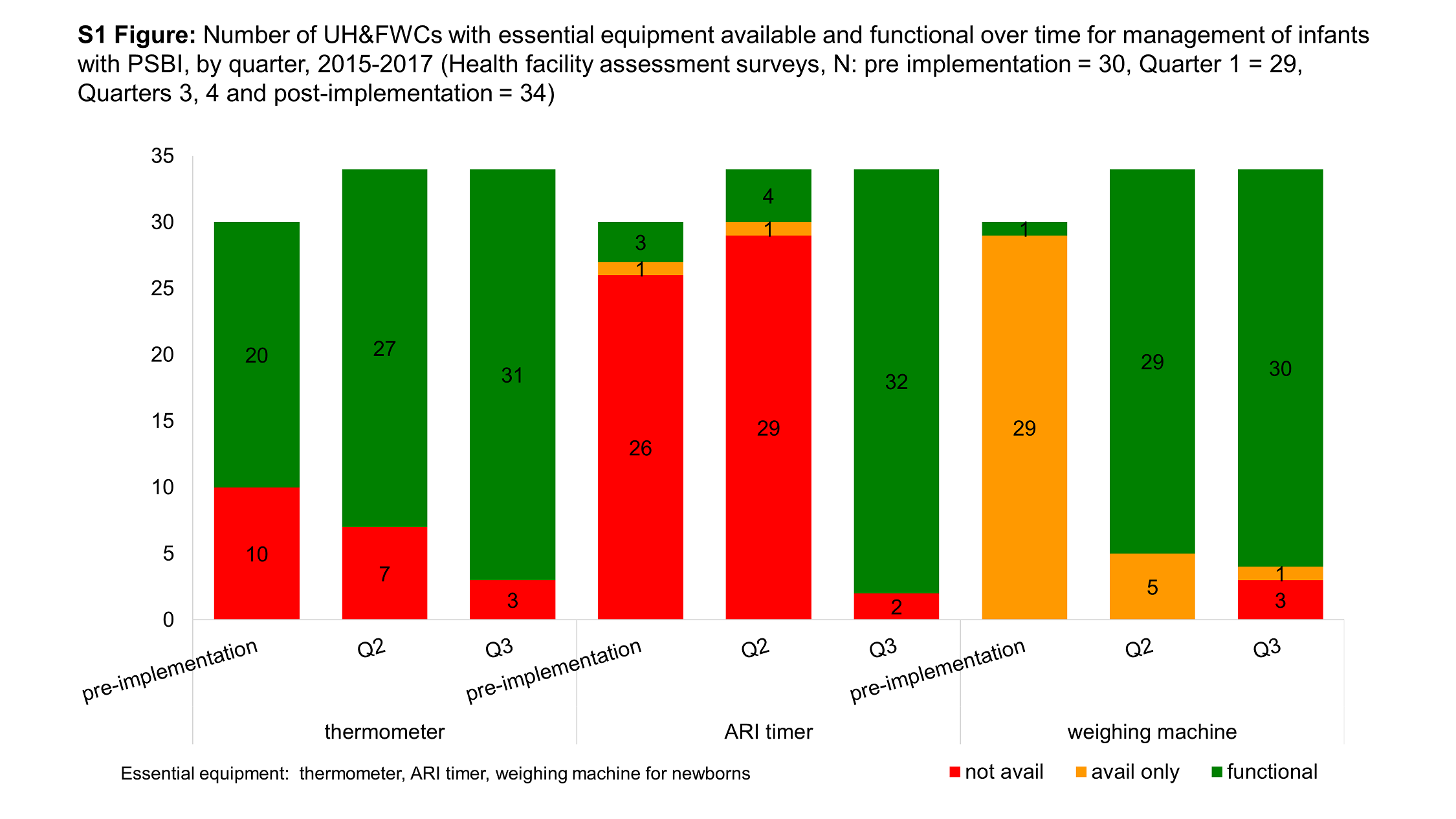

Supplement: S1 Fig — (TIF) [file pone.0232675.s007.tif]

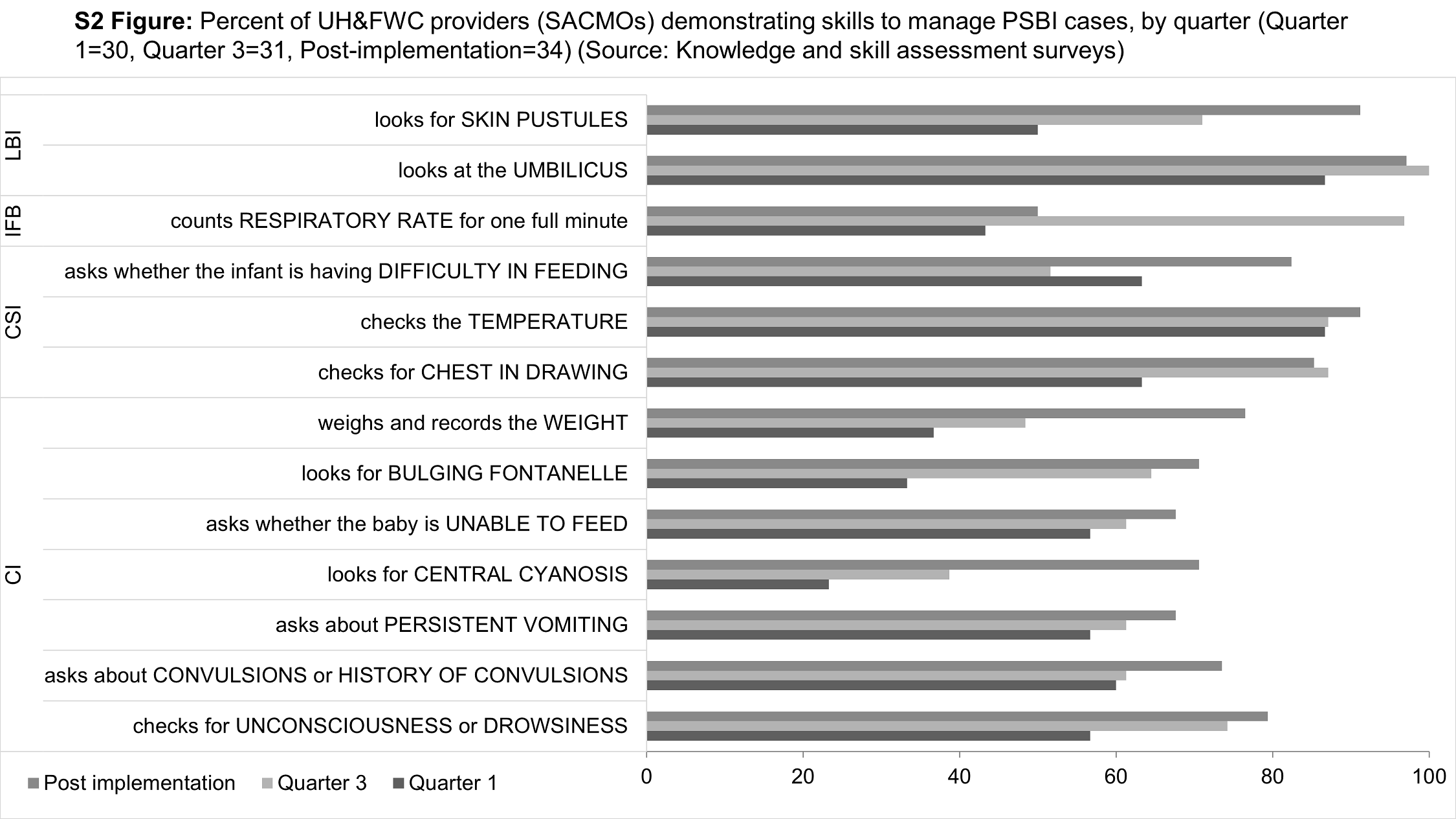

Supplement: S2 Fig — (TIF) [file pone.0232675.s008.tif]

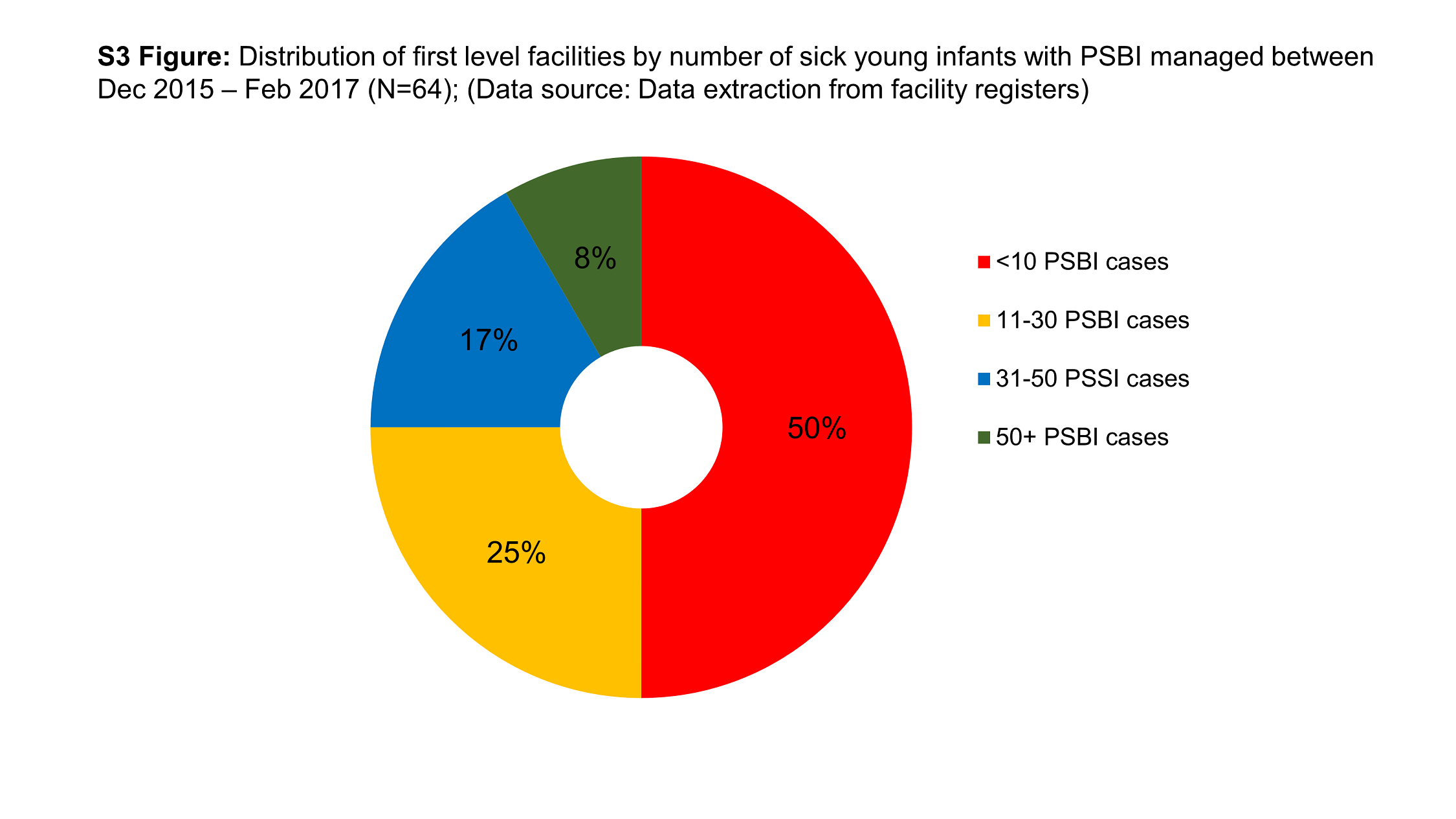

Supplement: S3 Fig — (TIF) [file pone.0232675.s009.tif]

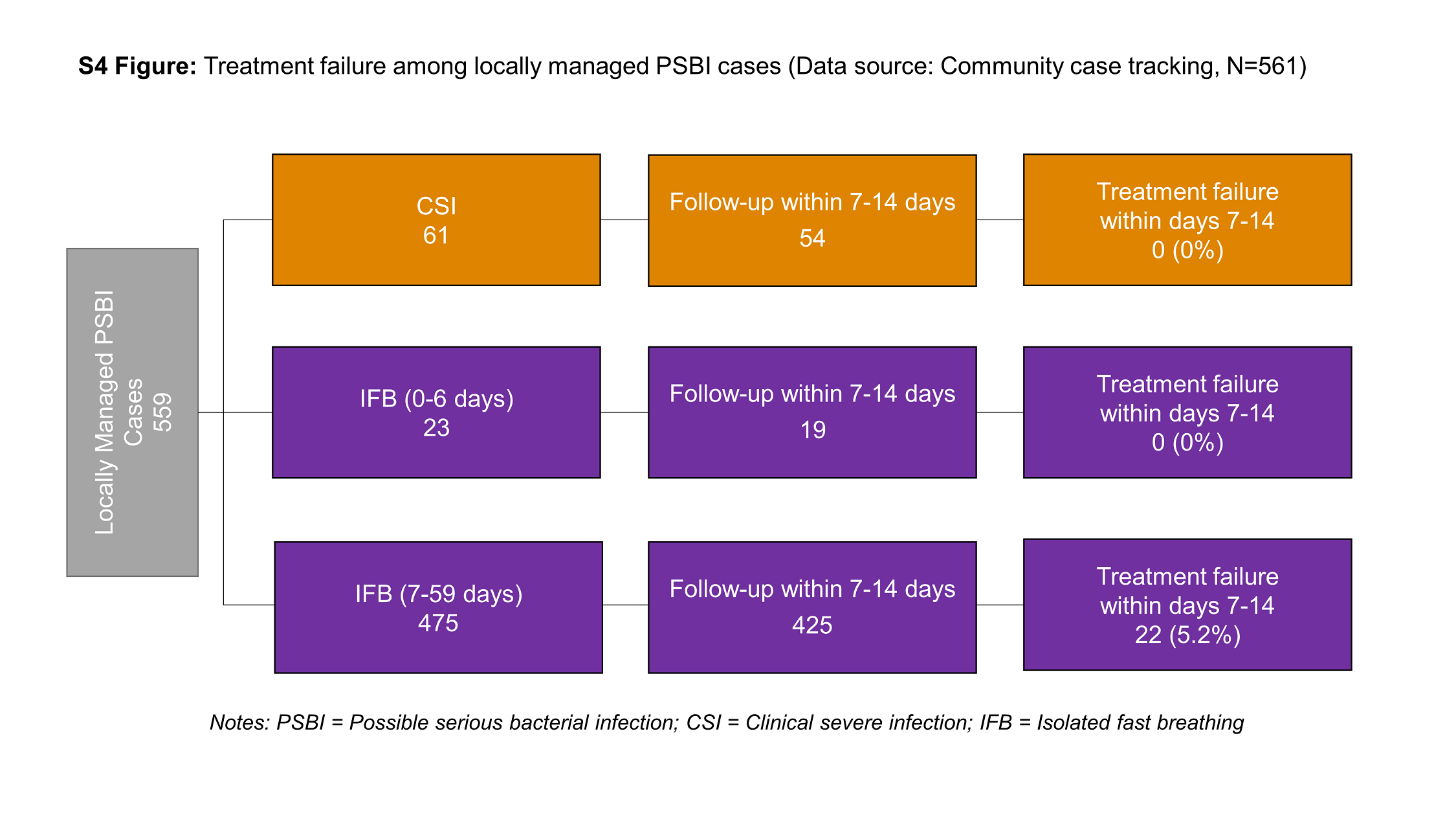

Supplement: S4 Fig — (TIF) [file pone.0232675.s010.tif]

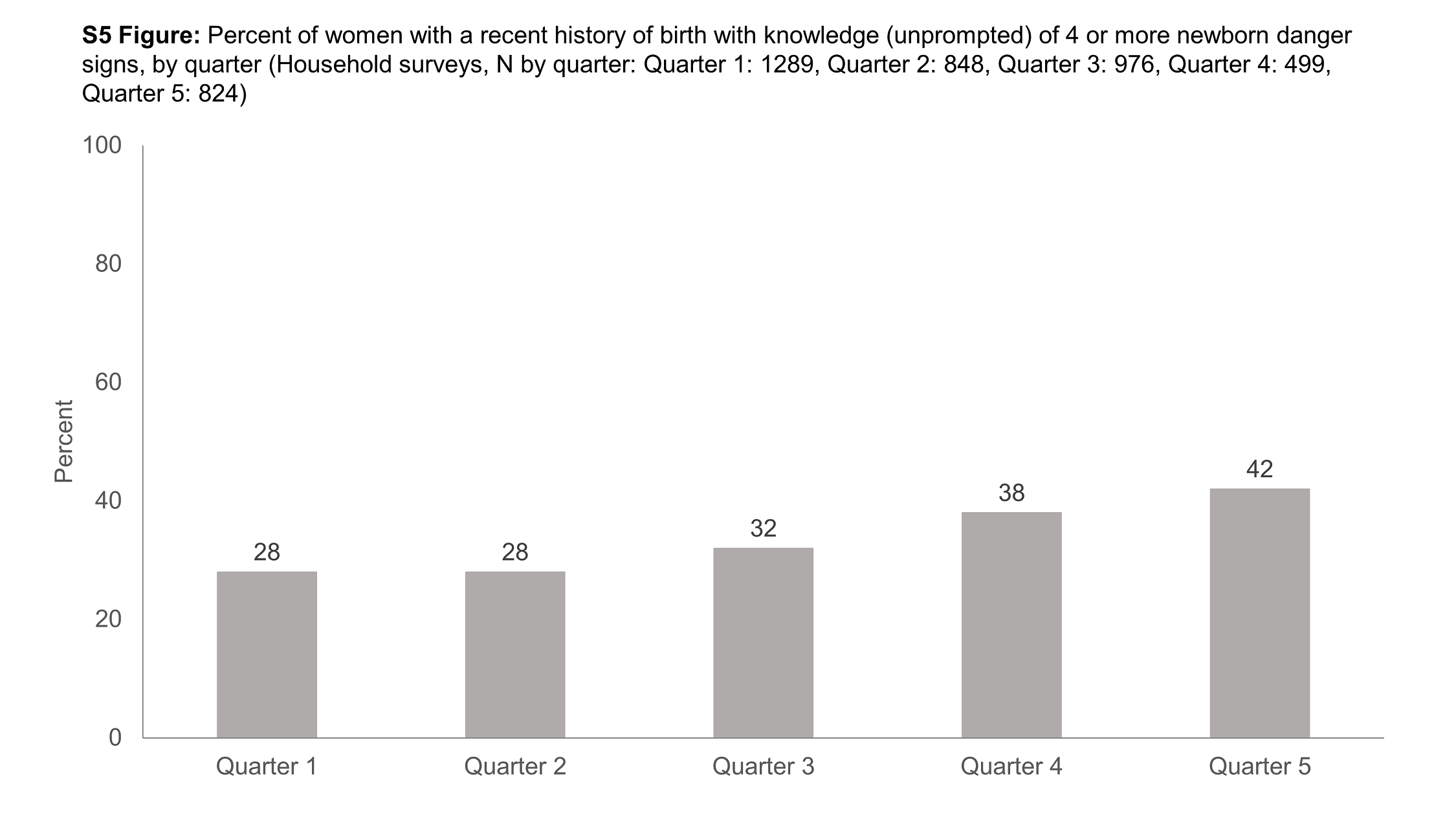

Supplement: S5 Fig — (TIF) [file pone.0232675.s011.tif]
